# 5-Acetyl-4-(4-chloro­phen­yl)-6-methyl-3,4-dihydro­pyrimidine-2(1*H*)-thione

**DOI:** 10.1107/S1600536809005029

**Published:** 2009-02-21

**Authors:** N. Anuradha, A. Thiruvalluvar, K. Pandiarajan, S. Chitra, R. J. Butcher

**Affiliations:** a PG Research Department of Physics, Rajah Serfoji Government College (Autonomous), Thanjavur 613 005, Tamil Nadu, India; b Department of Chemistry, Annamalai University, Annamalai Nagar 608 002, Tamilnadu, India; c Department of Chemistry, Howard University, 525 College Street NW, Washington, DC 20059, USA

## Abstract

In the title mol­ecule, C_13_H_13_ClN_2_OS, the heterocyclic ring adopts a flattened boat conformation, and the plane through the four coplanar atoms makes a dihedral angle of 87.92 (10)° with the benzene ring. The thione, acetyl and methyl groups have equatorial orientations with respect to the attached heterocyclic ring. The chloro­phenyl group has an axial orientation. Inter­molecular N—H⋯O, N—H⋯S and C—H⋯O hydrogen bonds are found in the crystal structure.

## Related literature

For dihydro­pyrimidin-2(1*H*)-ones as anti-oxidant agents, see: Stefani *et al.* (2006 ▶), and for their biological activity, see: Patil *et al.* (1995 ▶). For dihydro­pyrimidinones as calcium channel blockers, see: Rovnyak *et al.* (1995 ▶); Atwal *et al.* (1990 ▶) and as anti­hypertensive agents, see: Atwal *et al.* (1991 ▶); Grover *et al.* (1995 ▶). For the biological activity of marine alkaloids possessing a dihydro­pyrimidine-5-carboxyl­ate core, see: Patil *et al.* (1995 ▶). For the biological activity of dihydropyrimidin-2(1*H*)-thiones, see: Kappe (1993 ▶).
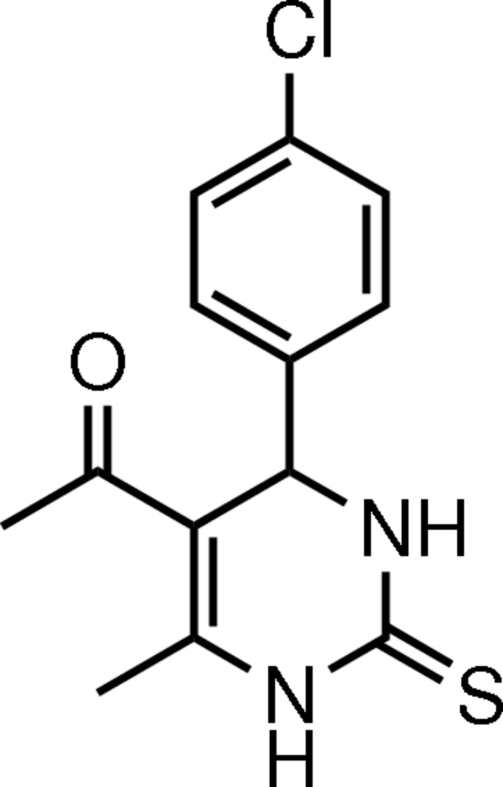


## Experimental

### 

#### Crystal data

C_13_H_13_ClN_2_OS
*M* *_r_* = 280.77
Triclinic, 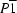

*a* = 7.2389 (6) Å
*b* = 8.2304 (7) Å
*c* = 12.9038 (11) Å
α = 73.366 (7)°
β = 89.373 (7)°
γ = 72.613 (7)°
*V* = 700.62 (11) Å^3^
*Z* = 2
Cu *K*α radiation
μ = 3.72 mm^−1^
*T* = 295 K
0.42 × 0.25 × 0.22 mm

#### Data collection

Oxford Diffraction Gemini R diffractometer
Absorption correction: multi-scan (*CrysAlis RED*; Oxford Diffraction, 2008 ▶) *T*_min_ = 0.182, *T*_max_ = 1.000 (expected range = 0.080–0.441)
6666 measured reflections
2878 independent reflections
2105 reflections with *I* > 2σ(*I*)
*R*_int_ = 0.036

#### Refinement

*R*[*F* ^2^ > 2σ(*F* ^2^)] = 0.071
*wR*(*F* ^2^) = 0.202
*S* = 1.03
2878 reflections
173 parameters
H atoms treated by a mixture of independent and constrained refinement
Δρ_max_ = 0.53 e Å^−3^
Δρ_min_ = −0.23 e Å^−3^

### 

Data collection: *CrysAlis CCD* (Oxford Diffraction, 2008 ▶); cell refinement: *CrysAlis RED* (Oxford Diffraction, 2008 ▶); data reduction: *CrysAlis RED*; program(s) used to solve structure: *SHELXS97* (Sheldrick, 2008 ▶); program(s) used to refine structure: *SHELXL97* (Sheldrick, 2008 ▶); molecular graphics: *ORTEP-3* (Farrugia, 1997 ▶); software used to prepare material for publication: *PLATON* (Spek, 2009 ▶).

## Supplementary Material

Crystal structure: contains datablocks global, I. DOI: 10.1107/S1600536809005029/hg2476sup1.cif

Structure factors: contains datablocks I. DOI: 10.1107/S1600536809005029/hg2476Isup2.hkl

Additional supplementary materials:  crystallographic information; 3D view; checkCIF report

## Figures and Tables

**Table 1:** Hydrogen-bond geometry (Å, °)

*D*—H⋯*A*	*D*—H	H⋯*A*	*D*⋯*A*	*D*—H⋯*A*
N1—H1⋯O15^i^	0.83 (4)	2.06 (4)	2.882 (3)	175 (4)
N3—H3⋯S2^ii^	0.90 (4)	2.43 (4)	3.328 (3)	172 (3)
C61—H61*B*⋯O15^i^	0.96	2.58	3.405 (4)	144

Symmetry codes: (i) 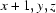; (ii) 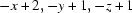.

**Fig. 1.**
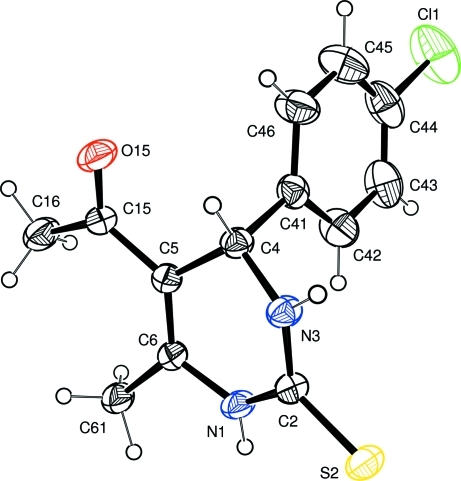
The molecular structure of the title compound, showing the atom-numbering scheme and displacement ellipsoids drawn at the 30% probability level. H atoms are shown as small spheres of arbitrary radius.

**Fig. 2.**
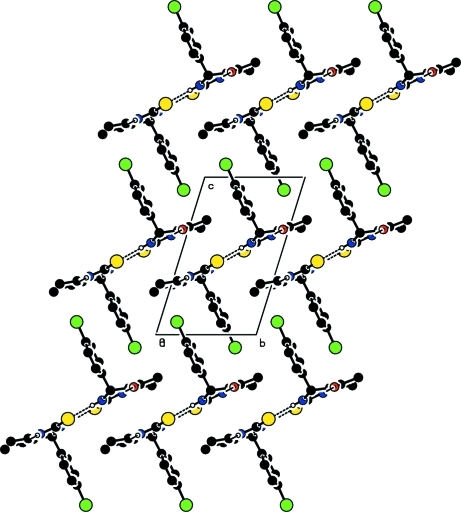
The packing of the title compound, viewed down the a axis. Dashed lines indicate hydrogen bonds. H atoms not involved in hydrogen bonding have been omitted.

## supplementary crystallographic information

### Comment

Dihydropyrimidin-2(1*H*)-ones are pharmacologically acitive as antioxidant agents (Stefani *et al.*, 2006). In recent years, much research has been focused on the synthesis of dihydropyrimidinones, which are important compounds due to their therapeutic and pharmacological properties. For example, they can serve as the integral of several calcium channel blockers (Rovnyak *et al.*, 1995; Atwal *et al.*, 1990), antihypertensive agents (Atwal *et al.*, 1991; Grover *et al.*, 1995). Recently, some marine alkaloids possessing dihydropyrimidine-5-carboxylate core have been shown to exhibit interesting biological activities such as potent HIV-gp-120-CD4 inhibitors as well as anti-HIV agents (Patil *et al.*, 1995). Dihydropyrimidin-2(1*H*)-thiones are also of much interest with regard to biological activity (Kappe, 1993).

In the title molecule, C_13_H_13_ClN_2_OS, Fig.1., the heterocyclic ring adopts a flattened boat conformation, and the plane through the four coplanar atoms(C2,N3,C5 and C6) makes a dihedral angle of 87.92 (10)° with the benzene ring. The thione, acetyl and methyl groups have equatorial orientation, with the attached heterocyclic ring. The chlorophenyl group has an axial orientation. N1—H1···O15(1 + *x*, *y*, *z*), N3—H3···S2(2 - *x*, 1 - *y*, 1 - *z*) and C61—H61B···O15(1 + *x*, *y*, *z*) intermolecular hydrogen bonds are found in the crystal structure(Fig.2., Table 1).

### Experimental

A solution of acetylacetone (1.0012 g, 0.01 mol), 4-chlorobenzaldehyde (1.40 g, 0.01 mol) and thiourea (1.14 g, 0.015 mol) was heated under reflux in the presence of calcium fluoride (0.07 g, 0.001 mol) for 2 h (monitored by TLC). After, completion of the reaction, the reaction mixture was cooled to room temperature and poured into crushed ice. The solid product was filtered under suction and purified by recrystallization from hot methanol to gave the product in the pure form. Yield 1.02 g (90%).

### Refinement

H1 at N1 and H3 at N3 atoms were located in a difference Fourier map and refined isotropically. Remaining H atoms were positioned geometrically and allowed to ride on their parent atoms, with C—H = 0.93 - 0.98 Å and *U*_iso_(H) = 1.2 - 1.5 times *U*_eq_(C).

### Crystal data

**Table:**

C_13_H_13_ClN_2_OS	*Z* = 2
*M**_r_* = 280.77	*F*(000) = 292
Triclinic, *P*1	*D*_x_ = 1.331 Mg m^−^^3^
Hall symbol: -P 1	Melting point: 529.5 K
*a* = 7.2389 (6) Å	Cu *K*α radiation, λ = 1.54184 Å
*b* = 8.2304 (7) Å	Cell parameters from 2326 reflections
*c* = 12.9038 (11) Å	θ = 5.9–77.2°
α = 73.366 (7)°	µ = 3.72 mm^−^^1^
β = 89.373 (7)°	*T* = 295 K
γ = 72.613 (7)°	Prism, colourless
*V* = 700.62 (11) Å^3^	0.42 × 0.25 × 0.22 mm

### Data collection

**Table:**

Oxford Diffraction Gemini R diffractometer	2878 independent reflections
Radiation source: fine-focus sealed tube	2105 reflections with *I* > 2σ(*I*)
graphite	*R*_int_ = 0.036
Detector resolution: 10.5081 pixels mm^-1^	θ_max_ = 77.4°, θ_min_ = 5.9°
φ and ω scans	*h* = −9→5
Absorption correction: multi-scan (*CrysAlis RED*; Oxford Diffraction, 2008)	*k* = −10→9
*T*_min_ = 0.182, *T*_max_ = 1.000	*l* = −16→16
6666 measured reflections	

### Refinement

**Table:**

Refinement on *F*^2^	Primary atom site location: structure-invariant direct methods
Least-squares matrix: full	Secondary atom site location: difference Fourier map
*R*[*F*^2^ > 2σ(*F*^2^)] = 0.071	Hydrogen site location: inferred from neighbouring sites
*wR*(*F*^2^) = 0.202	H atoms treated by a mixture of independent and constrained refinement
*S* = 1.03	*w* = 1/[σ^2^(*F*_o_^2^) + (0.1275*P*)^2^ + 0.1258*P*] where *P* = (*F*_o_^2^ + 2*F*_c_^2^)/3
2878 reflections	(Δ/σ)_max_ = 0.001
173 parameters	Δρ_max_ = 0.53 e Å^−^^3^
0 restraints	Δρ_min_ = −0.23 e Å^−^^3^

### Special details

**Table:**

Geometry. Bond distances, angles *etc*. have been calculated using the rounded fractional coordinates. All su's are estimated from the variances of the (full) variance-covariance matrix. The cell e.s.d.'s are taken into account in the estimation of distances, angles and torsion angles
Refinement. Refinement of *F*^2^ against ALL reflections. The weighted *R*-factor *wR* and goodness of fit *S* are based on *F*^2^, conventional *R*-factors *R* are based on *F*, with *F* set to zero for negative *F*^2^. The threshold expression of *F*^2^ > 2σ(*F*^2^) is used only for calculating *R*-factors(gt) *etc*. and is not relevant to the choice of reflections for refinement. *R*-factors based on *F*^2^ are statistically about twice as large as those based on *F*, and *R*- factors based on ALL data will be even larger.

### Fractional atomic coordinates and isotropic or equivalent isotropic displacement parameters (Å^2^)

**Table:**

	*x*	*y*	*z*	*U*_iso_*/*U*_eq_	
Cl1	0.5516 (3)	0.8292 (2)	−0.08141 (12)	0.1388 (8)	
S2	1.30462 (11)	0.38375 (11)	0.46485 (8)	0.0548 (3)	
O15	0.6554 (3)	0.0586 (3)	0.3317 (3)	0.0656 (9)	
N1	1.2495 (3)	0.1540 (3)	0.3701 (2)	0.0469 (8)	
N3	0.9758 (3)	0.3326 (3)	0.4163 (2)	0.0449 (8)	
C2	1.1665 (4)	0.2871 (4)	0.4144 (3)	0.0429 (8)	
C4	0.8486 (4)	0.2810 (4)	0.3534 (3)	0.0418 (8)	
C5	0.9546 (4)	0.1023 (4)	0.3372 (3)	0.0410 (8)	
C6	1.1509 (4)	0.0490 (4)	0.3417 (2)	0.0410 (8)	
C15	0.8251 (4)	0.0061 (4)	0.3157 (3)	0.0467 (9)	
C16	0.8904 (5)	−0.1503 (5)	0.2717 (4)	0.0669 (13)	
C41	0.7733 (5)	0.4234 (4)	0.2449 (3)	0.0486 (9)	
C42	0.8999 (6)	0.4851 (5)	0.1744 (3)	0.0681 (12)	
C43	0.8321 (8)	0.6118 (6)	0.0741 (4)	0.0832 (16)	
C44	0.6360 (9)	0.6762 (6)	0.0464 (4)	0.0843 (16)	
C45	0.5095 (8)	0.6201 (7)	0.1132 (5)	0.0967 (19)	
C46	0.5777 (6)	0.4951 (6)	0.2145 (4)	0.0738 (16)	
C61	1.2863 (4)	−0.1154 (4)	0.3226 (3)	0.0563 (12)	
H1	1.367 (5)	0.131 (4)	0.361 (3)	0.040 (8)*	
H3	0.910 (5)	0.415 (5)	0.448 (3)	0.058 (10)*	
H4	0.73685	0.26846	0.39490	0.0500*	
H16A	0.78061	−0.18685	0.25915	0.1003*	
H16B	0.98424	−0.24715	0.32324	0.1003*	
H16C	0.94799	−0.11707	0.20472	0.1003*	
H42	1.03260	0.44097	0.19439	0.0819*	
H43	0.91806	0.65179	0.02692	0.0998*	
H45	0.37713	0.66430	0.09217	0.1159*	
H46	0.48960	0.45990	0.26195	0.0886*	
H61A	1.25700	−0.21851	0.36724	0.0843*	
H61B	1.41785	−0.12402	0.34080	0.0843*	
H61C	1.27086	−0.10909	0.24764	0.0843*	

### Atomic displacement parameters (Å^2^)

**Table:**

	*U*^11^	*U*^22^	*U*^33^	*U*^12^	*U*^13^	*U*^23^
Cl1	0.2014 (19)	0.0993 (10)	0.0671 (8)	−0.0081 (12)	−0.0255 (10)	0.0105 (7)
S2	0.0423 (4)	0.0610 (5)	0.0724 (6)	−0.0201 (3)	0.0039 (3)	−0.0325 (4)
O15	0.0355 (11)	0.0666 (14)	0.105 (2)	−0.0201 (10)	0.0099 (12)	−0.0365 (14)
N1	0.0286 (11)	0.0521 (14)	0.0644 (17)	−0.0132 (10)	0.0056 (10)	−0.0232 (12)
N3	0.0377 (12)	0.0493 (13)	0.0526 (15)	−0.0123 (10)	0.0049 (10)	−0.0238 (12)
C2	0.0392 (14)	0.0462 (14)	0.0448 (16)	−0.0147 (12)	0.0011 (12)	−0.0141 (12)
C4	0.0353 (13)	0.0450 (14)	0.0465 (16)	−0.0120 (11)	0.0055 (11)	−0.0164 (12)
C5	0.0373 (13)	0.0388 (13)	0.0467 (16)	−0.0110 (10)	0.0029 (11)	−0.0133 (12)
C6	0.0368 (13)	0.0406 (13)	0.0465 (16)	−0.0135 (11)	0.0033 (11)	−0.0126 (11)
C15	0.0375 (14)	0.0459 (15)	0.0549 (18)	−0.0152 (12)	−0.0018 (12)	−0.0094 (13)
C16	0.0512 (18)	0.064 (2)	0.098 (3)	−0.0223 (16)	0.0000 (19)	−0.038 (2)
C41	0.0530 (16)	0.0433 (15)	0.0510 (18)	−0.0114 (12)	0.0036 (14)	−0.0200 (13)
C42	0.065 (2)	0.067 (2)	0.063 (2)	−0.0151 (18)	0.0140 (18)	−0.0108 (18)
C43	0.114 (4)	0.070 (2)	0.059 (2)	−0.027 (3)	0.019 (2)	−0.011 (2)
C44	0.120 (4)	0.059 (2)	0.054 (2)	−0.006 (2)	−0.010 (2)	−0.0086 (18)
C45	0.081 (3)	0.095 (4)	0.082 (3)	0.000 (3)	−0.025 (3)	−0.005 (3)
C46	0.055 (2)	0.078 (3)	0.069 (3)	−0.0081 (18)	−0.0066 (18)	−0.005 (2)
C61	0.0390 (15)	0.0486 (16)	0.085 (3)	−0.0129 (12)	0.0042 (15)	−0.0259 (16)

### Geometric parameters (Å, °)

**Table:**

Cl1—C44	1.747 (5)	C41—C42	1.386 (6)
S2—C2	1.686 (3)	C42—C43	1.392 (6)
O15—C15	1.211 (4)	C43—C44	1.370 (9)
N1—C2	1.359 (4)	C44—C45	1.343 (9)
N1—C6	1.395 (4)	C45—C46	1.397 (8)
N3—C2	1.320 (4)	C4—H4	0.9800
N3—C4	1.461 (4)	C16—H16A	0.9600
N1—H1	0.83 (4)	C16—H16B	0.9600
N3—H3	0.90 (4)	C16—H16C	0.9600
C4—C41	1.525 (5)	C42—H42	0.9300
C4—C5	1.515 (5)	C43—H43	0.9300
C5—C6	1.353 (4)	C45—H45	0.9300
C5—C15	1.469 (4)	C46—H46	0.9300
C6—C61	1.498 (5)	C61—H61A	0.9600
C15—C16	1.501 (5)	C61—H61B	0.9600
C41—C46	1.374 (6)	C61—H61C	0.9600
			
Cl1···O15^i^	3.332 (4)	C16···H61C	2.8800
S2···N3^ii^	3.328 (3)	C16···H61A	2.7700
S2···H46^iii^	2.9200	C42···H16A^iv^	3.0800
S2···H61A^iv^	3.0700	C43···H43^viii^	2.9800
S2···H4^iii^	3.1900	C61···H16B	2.7100
S2···H3^ii^	2.43 (4)	C61···H16C	2.9000
S2···H61B^v^	3.0600	H1···O15^iii^	2.06 (4)
O15···N1^vi^	2.882 (3)	H1···H61B	2.1100
O15···C41	3.253 (4)	H3···S2^ii^	2.43 (4)
O15···C46	3.348 (6)	H4···S2^vi^	3.1900
O15···C61^vi^	3.405 (4)	H4···O15	2.3300
O15···Cl1^i^	3.332 (4)	H4···H46	2.3400
O15···H4	2.3300	H16A···C42^vii^	3.0800
O15···H61B^vi^	2.5800	H16B···C6	3.0900
O15···H1^vi^	2.06 (4)	H16B···C61	2.7100
N1···O15^iii^	2.882 (3)	H16B···H61A	2.1600
N3···S2^ii^	3.328 (3)	H16C···C61	2.9000
N1···H42	2.8200	H16C···H61C	2.4300
N3···H42	2.8100	H42···N1	2.8200
C2···C42	3.366 (5)	H42···N3	2.8100
C6···C42	3.549 (5)	H42···C2	2.8100
C16···C61	3.061 (5)	H42···C5	3.0800
C16···C42^vii^	3.552 (6)	H43···C43^viii^	2.9800
C41···O15	3.253 (4)	H46···S2^vi^	2.9200
C42···C6	3.549 (5)	H46···H4	2.3400
C42···C2	3.366 (5)	H61A···S2^vii^	3.0700
C42···C16^iv^	3.552 (6)	H61A···C15	3.0900
C43···C43^viii^	3.492 (7)	H61A···C16	2.7700
C46···O15	3.348 (6)	H61A···H16B	2.1600
C61···C16	3.061 (5)	H61B···O15^iii^	2.5800
C61···O15^iii^	3.405 (4)	H61B···H1	2.1100
C2···H42	2.8100	H61B···S2^v^	3.0600
C5···H42	3.0800	H61C···C16	2.8800
C6···H16B	3.0900	H61C···H16C	2.4300
C15···H61A	3.0900		
			
C2—N1—C6	124.2 (2)	C43—C44—C45	121.7 (5)
C2—N3—C4	124.3 (3)	Cl1—C44—C45	119.8 (5)
C2—N1—H1	118 (2)	C44—C45—C46	119.7 (5)
C6—N1—H1	118 (2)	C41—C46—C45	120.8 (4)
C2—N3—H3	122 (2)	N3—C4—H4	108.00
C4—N3—H3	113 (2)	C5—C4—H4	108.00
S2—C2—N3	123.2 (3)	C41—C4—H4	108.00
N1—C2—N3	116.3 (3)	C15—C16—H16A	109.00
S2—C2—N1	120.5 (2)	C15—C16—H16B	109.00
N3—C4—C5	110.0 (3)	C15—C16—H16C	110.00
N3—C4—C41	111.2 (3)	H16A—C16—H16B	109.00
C5—C4—C41	111.1 (3)	H16A—C16—H16C	110.00
C4—C5—C15	113.8 (3)	H16B—C16—H16C	110.00
C6—C5—C15	127.1 (3)	C41—C42—H42	119.00
C4—C5—C6	119.1 (3)	C43—C42—H42	119.00
N1—C6—C5	118.6 (3)	C42—C43—H43	121.00
N1—C6—C61	112.4 (3)	C44—C43—H43	121.00
C5—C6—C61	129.0 (3)	C44—C45—H45	120.00
C5—C15—C16	123.5 (3)	C46—C45—H45	120.00
O15—C15—C5	118.3 (3)	C41—C46—H46	120.00
O15—C15—C16	118.2 (3)	C45—C46—H46	120.00
C4—C41—C46	120.9 (3)	C6—C61—H61A	109.00
C42—C41—C46	118.0 (4)	C6—C61—H61B	109.00
C4—C41—C42	121.1 (3)	C6—C61—H61C	109.00
C41—C42—C43	121.2 (4)	H61A—C61—H61B	109.00
C42—C43—C44	118.5 (5)	H61A—C61—H61C	109.00
Cl1—C44—C43	118.5 (4)	H61B—C61—H61C	109.00
			
C6—N1—C2—S2	169.9 (2)	C4—C5—C6—C61	−176.4 (3)
C6—N1—C2—N3	−9.4 (5)	C15—C5—C6—N1	−176.0 (3)
C2—N1—C6—C5	13.7 (4)	C15—C5—C6—C61	2.4 (6)
C2—N1—C6—C61	−164.9 (3)	C4—C5—C15—O15	−12.9 (5)
C4—N3—C2—S2	166.0 (3)	C4—C5—C15—C16	165.2 (4)
C4—N3—C2—N1	−14.8 (5)	C6—C5—C15—O15	168.3 (4)
C2—N3—C4—C5	30.5 (4)	C6—C5—C15—C16	−13.7 (6)
C2—N3—C4—C41	−93.1 (4)	C4—C41—C42—C43	178.4 (4)
N3—C4—C5—C6	−24.4 (4)	C46—C41—C42—C43	−2.0 (6)
N3—C4—C5—C15	156.7 (3)	C4—C41—C46—C45	−177.3 (4)
C41—C4—C5—C6	99.1 (4)	C42—C41—C46—C45	3.1 (7)
C41—C4—C5—C15	−79.8 (4)	C41—C42—C43—C44	0.6 (7)
N3—C4—C41—C42	53.6 (4)	C42—C43—C44—Cl1	−177.8 (4)
N3—C4—C41—C46	−126.0 (4)	C42—C43—C44—C45	−0.1 (8)
C5—C4—C41—C42	−69.3 (4)	Cl1—C44—C45—C46	178.7 (4)
C5—C4—C41—C46	111.1 (4)	C43—C44—C45—C46	1.1 (8)
C4—C5—C6—N1	5.3 (5)	C44—C45—C46—C41	−2.7 (8)

Symmetry codes: (i) −*x*+1, −*y*+1, −*z*; (ii) −*x*+2, −*y*+1, −*z*+1; (iii) *x*+1, *y*, *z*; (iv) *x*, *y*+1, *z*; (v) −*x*+3, −*y*, −*z*+1; (vi) *x*−1, *y*, *z*; (vii) *x*, *y*−1, *z*; (viii) −*x*+2, −*y*+1, −*z*.

### Hydrogen-bond geometry (Å, °)

**Table:**

*D*—H···*A*	*D*—H	H···*A*	*D*···*A*	*D*—H···*A*
N1—H1···O15^iii^	0.83 (4)	2.06 (4)	2.882 (3)	175 (4)
N3—H3···S2^ii^	0.90 (4)	2.43 (4)	3.328 (3)	172 (3)
C61—H61B···O15^iii^	0.96	2.58	3.405 (4)	144

Symmetry codes: (iii) *x*+1, *y*, *z*; (ii) −*x*+2, −*y*+1, −*z*+1.
